# LncRNA ALMS1-IT1 is a novel prognostic biomarker and correlated with immune infiltrates in colon adenocarcinoma

**DOI:** 10.1097/MD.0000000000031314

**Published:** 2022-10-21

**Authors:** Yuning Lin, Ying Li, Yongquan Chen, Zhongying Zhang

**Affiliations:** a Xiamen Key Laboratory of Biomarker Translational Medicine, Medical Laboratory of Xiamen Humanity Hospital Fujian Medical University, Xiamen, China; b Ultrasonography Department, Women and Children’s Hospital, School of Medicine, Xiamen University, Xiamen, China.

**Keywords:** ALMS1-IT1, biomarker, LncRNA

## Abstract

Colon adenocarcinoma (COAD) is one of the most serious cancers. It is important to accurately predict prognosis and provide individualized treatment. Evidence suggests that clinicopathological features and immune status of the body are related to the occurrence and development of cancer. Expression of long non-coding RNA (LncRNA) ALMS1 intronic transcript 1 (ALMS1-IT1) is observed in some cancer types, and we believe that it may have the potential to serve as a marker of COAD. Therefore, we used the data obtained from the cancer genome atlas (TCGA) database to prove the relationship between ALMS1-IT1 and COAD. Wilcoxon rank sum test, Chi-square test, Fisher exact test and logistic regression were used to evaluate relationships between clinical-pathologic features and ALMS1-IT1 expression. Receiver operating characteristic curves were used to describe binary classifier value of ALMS1-IT1 using area under curve score. Kaplan–Meier method and Cox regression analysis were used to evaluate factors contributing to prognosis. Gene oncology (GO) and (Kyoto Encyclopedia of Genes and Genomes) KEGG enrichment analysis were used to predict the function of differentially expressed genes associated with ALMS1-IT1. Gene set enrichment analysis (GSEA) was used to predict canonical pathways associated with ALMS1-IT1.Immune infiltration analysis was performed to identify the significantly involved functions of ALMS1-IT1. Starbase database was used to predict miRNAs and RNA binding proteins (RBPs) that may interact with ALMS1-IT1. Increased ALMS1-IT1 expression in COAD was associated with N stage (*P* < .001), M stage (*P* = .003), Pathologic stage (*P* = .002), and Primary therapy outcome (*P* = .009). Receiver operating characteristic curve suggested the significant diagnostic and prognostic ability of ALMS1-IT1 (area under curve = 0.857). High ALMS1-IT1 expression predicted a poorer overall-survival (*P* = .005) and poorer progression-free interval (PFI) (*P* = .012), and ALMS1-IT1 expression was independently correlated with PFI in COAD patients (hazard ratio (HR) :1.468; 95% CI: 1.029–2.093; P =.034) (HR: 1.468; 95% CI: 1.029–2.093; *P* = .034). GO, KEGG, GSEA, and immune infiltration analysis showed that ALMS1-IT1 expression was correlated with regulating the function of DNA and some types of immune infiltrating cells. ALMS1-IT1 expression was significantly correlated with poor survival and immune infiltrations in COAD, and it may be a promising prognostic biomarker in COAD.

## 1. Introduction

Colorectal cancer (CRC) includes colon adenocarcinoma (COAD) and rectal adenocarcinoma, according to pathological classification, approximately 80% to 90% of CRC cases are COAD.^[1]^ CRC ranks third in terms of incidence (10.2% of total cases) and is the second leading cause of cancer-related death globally (9.2% of all cases).^[2]^ In the past few decades, the incidence and mortality of CRC have steadily declined globally, however, CRC is still the most common gastrointestinal malignancy and the second largest cancer-related disease cause of death.^[3]^ The use of chemotherapy and surgical resection for malignant CRC is increasing, but the effect of these treatments has not been significantly improved. About half of CRC recur and patients die within 5 years.^[4,5]^ Therefore, it is necessary to identify new diagnostic, prognostic biomarkers and therapeutic targets, as well as to study the potential molecular mechanisms of COAD. Good diagnostic and prognostic biomarkers should be closely related to the prognosis of patients and easy to detect.

Encouragingly, a large amount of evidence indicates that the regulatory role of long non-coding RNAs (LncRNAs) is related to the development and progression of a variety of cancers.^[6]^ LncRNAs are ≥200 nucleotides in length and do not encode proteins. According to its position and background in the genome, lncRNA can be divided into 5 main types: intergenic lncRNAs, intragenic lncRNAs, bidirectional lncRNAs, sense lncRNAs and antisense lncRNAs.^[7]^ The mechanisms of lncRNA regulating gene expression mainly include transcriptional repression, RNA-DNA interaction (chromatin remodeling), nuclear RNA-RNA interaction and cytoplasmic RNA-RNA interaction. Their functions are to regulate a series of cellular biological processes, including chromatin remodeling, transcriptional and post-transcriptional events.^[8,9]^ The most recognized molecular mechanism of lncRNAs is to act as a miRNA “sponge” to regulate downstream target genes.^[10,11]^ LncRNAs is abnormally expressed in various types of cancer cells and plays an important role in several common hallmarks of cancer.^[12]^ In addition, a growing number of studies indicate that lncRNAs may be identified as novel biomarkers for diagnosis, prognosis and metastasis prediction in various cancers.^[13–15]^ These year, experiments have demonstrated that several lncRNAs are CRC-specific lncRNAs, such as PCAT-1, RP11-462C24.1, HOTAIR, and MALAT1 as candidate diagnostic biomarkers.^[16–18]^

ALMS1-IT1,officical full name is ALMS1 intronic transcript 1. Up to now, there are few studies on ALMS1-IT1, and some studies believe that ALMS1-IT1 has prognostic value.^[19]^ Recent studies have predicted that the expression of ALMS1-IT1 may be related to ferroptosis.^[20]^ Bioinformatics analysis predicts that ALMS1-IT1 can serve as a prognostic biomarker for Head and neck squamous cell carcinoma (HNSCC).^[21]^ Experiments have shown that ALMS1-IT1/AVL9 promotes the malignant progression of lung adenocarcinoma (LUAD) in part by regulating the cyclin-dependent kinase pathway.^[22]^ Based on previous research results, we believe that ALMS1-IT1 may play an important role in the occurrence and development of COAD. Meanwhile, the role of ALMS1-IT1 in COAD has not been reported. Hence, in this research, we used the COAD RNA-seq data in the cancer genome atlas (TCGA) database to compare the difference of ALMS1-IT1 expression between tumor tissues and normal samples, and investigated the correlation between ALMS1-IT1 expression levels and clinical pathological features of COAD. Next, we evaluated the prognostic value of ALMS1-IT1 in COAD. In addition, gene oncology (GO), Kyoto Encyclopedia of Genes and Genomes (KEGG) and gene set enrichment analysis (GSEA) were performed on the high and low expression groups of ALMS1-IT1 to reveal its possible functions. Meanwhile, Starbase database was used to predict miRNAs and RNA binding proteins (RBPs) that may interact with ALMS1-IT1. Finally, by analyzing the correlation between ALMS1-IT1 expression and immune infiltration, we comprehensively explored and discussed the potential mechanism of ALMS1-IT1 regulating the occurrence and development of COAD.

## 2. Methods

### 2.1. RNA-sequencing data and bioinformatics analysis

We used TCGA database (https://portal.gdc.cancer.gov/) to collect RNA-seq data and clinical information from 521 cases of COAD projects, including 41 cases with matched adjacent tissues. The downloaded data format was level 3 HTSeq-fragments per kilobase per million and then was converted into transcripts per million format for subsequent analysis. We also download transcripts per million format RNA-seq data in TCGA and Genotype-Tissue Expression database that uniformly processed by Toil process from University of California Santa Cruz Xena (https://xenabrowser.net/datapages/).^[23^^]^ All procedures performed in this study were in accordance with the Declaration of Helsinki (as revised in 2013).

We used R package (DESeq2)^[24]^ to go differential analysis of ALMS1-IT1 expression, adjusted *P* value < 0.05 and |logFC| > 2 were consider as cut off criteria, the Different Expression Genes (DEGs) obtained were used for GO, KEGG analysis, adjusted *P* value <.05 were consider as another cut off criteria, the DEGs obtained were used for GSEA.

The R(version 3.6.0) package org. Hs.e.g..db(3.10.0) was used to conversion gene ID, cluster Profiler(3.14.3) was used to perform GO, KEGG, and GSEA between high- and low-ALMS1-IT1 groups.^[25,26]^ According to the default statistical method, the process was repeated 1000 times for each analysis and selected c2.cp.v7.2.symbols.gmt in MSigDB Collections as the reference gene collection, false discovery rate *q*-value < 0.25 and adjusted *P* adjust <.05 were considered to be significantly enriched.

### 2.2. Immune infiltration analysis by ssGSEA

The immune infiltration analysis of COAD was performed by single sample GSEA (ssGSEA) method from R (v.3.6.3) package GSVA (version 1.34.0),^[27]^ and we quantified the infiltration levels of 24 immune cell types from gene expression profile in the literature.^[28]^ In order to discover the correlation between ALMS1-IT1 and the infiltration levels of 24 immune cells, *P* values were determined by the Pearson and Wilcoxon rank sum test.

### 2.3. Target miRNA and protein prediction

Starbase database (https://starbase.sysu.edu.cn/) was used to predict miRNAs and RBPs that may interact with ALMS1-IT1.

### 2.4. Statistical analysis

All statistical analyses were performed using R(v.3.6.3). Wilcoxon rank sum test, chi square test, Fisher exact test and logistic regression were used to analyze the relationship between clinical pathologic features and ALMS1-IT1. Kaplan–Meier method was used to calculate the overall survival rate and progression-free interval (PFI) of COAD patients from TCGA. Univariate and multivariate analysis were performed to estimate the association between clinical and genetic clinical characteristics and PFI using Cox proportional hazard models. *P* values <.05 were considered statistically significant.

### 2.5. Ethical approval

This study does not involve experiments that require ethical approval.

## 3. Results

### 3.1. ALMS1-IT1 expression is correlated with poor clinicopathological features of COAD

**Figure 1.**
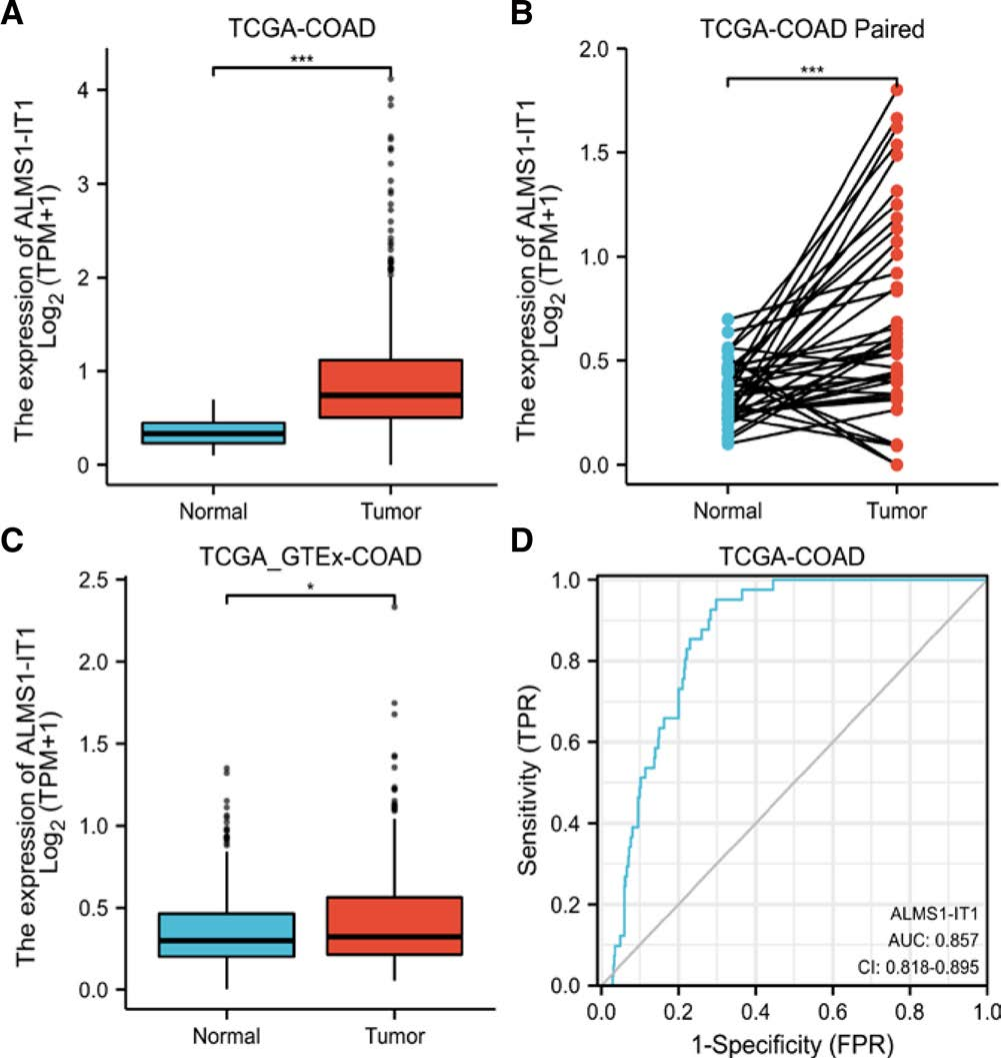
(A) Wilcoxon rank sum test was used to analyze the difference expression of ALMS1-IT1 in COAD tissues and adjacent colon tissues. (B) Wilcoxon signed rank sum test was used to detect the difference expression of ALMS1-IT1 in COAD tissues and adjacent colon tissues. (C) Wilcoxon rank sum test was used to analyze the difference expression of ALMS1-IT1 in normal colon tissues of GTEx combined with TCGA and COAD tissues of TCGA. (D) ROC curve showed the efficiency of ALMS1-IT1 expression level to distinguishing COAD tissue from non-tumor tissue. X-axis represents false positive rate, and Y-axis represents true positive rate. ALMS1-IT1 = ALMS1 intronic transcript 1, COAD = colon adenocarcinoma, GTEx = genotype-tissue expression, ROC = receiver operating characteristic, TCGA = the cancer genome atlas.

In order to identify the difference of ALMS1-IT1 expression between COAD and normal tissues, we analyzed the expression level of ALMS1-IT1 in 480 COAD tissues and 41 adjacent normal colon tissues, and found that ALMS1-IT1 was highly expressed in COAD tissues (*P* < .001, Fig. 1A). Meanwhile, we also analyzed the expression of ALMS1-IT1 in 41 COAD tissues and their matched adjacent tissues. The results indicated that COAD tissues highly expressed ALMS1-IT1 (*P* < .001, Fig. 1B). Moreover, the expression of ALMS1-IT1 in normal samples of Genotype-Tissue Expression combined TCGA database and COAD samples of TCGA database was compared. We also found that ALMS1-IT1 was significantly overexpressed in COAD samples (*P* = .037, Fig. 1C). In addition, we used the receiver operating characteristic curve to analyze the effectiveness of ALMS1-IT1 expression level to distinguish COAD tissues from non-tumor tissues. The area under curve of ALMS1-IT1 was 0.857, suggesting that ALMS1-IT1 could be served as an ideal biomarker to distinguish COAD from non-tumor tissue (Fig. 1D).

The characteristics of patients were shown in Table 1, in which 478 primary COAD with both clinical and gene expression data were collected from TCGA database. According to the mean value of relative ALMS1-IT1 expression, the patients with COAD were divided into high (n = 239) and low (n = 239) expression groups. The association between the expression level of ALMS1-IT1 and the clinicopathological characteristics of COAD patients was evaluated. Chi-square test or Fisher’s exact test revealed that ALMS1-IT1 expression was associated with N stage (*P* < .001), Gleason score (*P* = .002), primary therapy outcome (*P* = .001) and residual tumor (*P* < .001). Logistic regression method was also used to show the relationship between the clinicopathological characteristics of COAD and expression level of ALMS1-IT1. The results suggested that ALMS1-IT1 was significantly related to N stage (*P* < .001), M stage (*P* = .003), Pathologic stage (*P* = .002), and Primary therapy outcome (*P* = .009).

**Table 1:** Correlation between ALMS1-IT1 expression and clinicopathological characteristics in COAD.

Characteristic	Level	Low expression of ALMS1-IT1	High expression of ALMS1-IT1	*P*	Method
n		239	239		
T stage, n (%)				.107	Chisq.test
	T1	6 (1.3%)	5 (1%)		
	T2	49 (10.3%)	34 (7.1%)		
	T3	160 (33.5%)	163 (34.2%)		
	T4	23 (4.8%)	37 (7.8%)		
N stage, n (%)				.001	Chisq.test
	N0	160 (33.5%)	124 (25.9%)		
	N1	49 (10.3%)	59 (12.3%)		
	N2	30 (6.3%)	56 (11.7%)		
M stage, n (%)				.003	Chisq.test
	M0	194 (46.7%)	155 (37.3%)		
	M1	23 (5.5%)	43 (10.4%)		
Pathologic stage, n (%)				.002	Chisq.test
	Stage I	50 (10.7%)	31 (6.6%)		
	Stage II	104 (22.3%)	83 (17.8%)		
	Stage III	58 (12.4%)	75 (16.1%)		
	Stage IV	23 (4.9%)	43 (9.2%)		
Primary therapy outcome, n (%)				.009	Fisher.test
	PD	9 (3.6%)	16 (6.4%)		
	SD	4 (1.6%)	0 (0%)		
	PR	4 (1.6%)	9 (3.6%)		
	CR	122 (48.8%)	86 (34.4%)		
Gender, n (%)				.647	Chisq.test
	Female	110 (23%)	116 (24.3%)		
	Male	129 (27%)	123 (25.7%)		
Race, n (%)				.268	Fisher.test
	Asian	7 (2.3%)	4 (1.3%)		
	Black or African American	29 (9.5%)	34 (11.1%)		
	White	94 (30.7%)	138 (45.1%)		
Age, n (%)				.926	Chisq.test
	<=65	98 (20.5%)	96 (20.1%)		
	>65	141 (29.5%)	143 (29.9%)		
CEA level, n (%)				.257	Chisq.test
	<=5	110 (36.3%)	86 (28.4%)		
	>5	52 (17.2%)	55 (18.2%)		
Residual tumor, n (%)				.280	Fisher.test
	R0	189 (50.5%)	157 (42%)		
	R1	1 (0.3%)	3 (0.8%)		
	R2	10 (2.7%)	14 (3.7%)		
Lymphatic invasion, n (%)				.418	Chisq.test
	NO	137 (31.6%)	129 (29.7%)		
	YES	79 (18.2%)	89 (20.5%)		
OS event, n (%)				<.001	Chisq.test
	Alive	203 (42.5%)	172 (36%)		
	Dead	36 (7.5%)	67 (14%)		
PFI event, n (%)				.010	Chisq.test
	Alive	188 (39.3%)	162 (33.9%)		
	Dead	51 (10.7%)	77 (16.1%)		
DSS event, n (%)				.151	Chisq.test
	Alive	210 (45.5%)	188 (40.7%)		
	Dead	27 (5.8%)	37 (8%)		
Neoplasm type, n (%)				1.000	Fisher.test
Colon adenocarcinoma		239 (50%)	239 (50%)		
Rectum adenocarcinoma		0 (0%)	0 (0%)		
Colon polyps present, n (%)				.301	Chisq.test
	NO	72 (28.9%)	90 (36.1%)		
	YES	32 (12.9%)	55 (22.1%)		
History of colon polyps, n (%)				.096	Chisq.test
	NO	125 (30.6%)	137 (33.6%)		
	YES	83 (20.3%)	63 (15.4%)		
Perineural invasion, n (%)				.330	Chisq.test
	NO	66 (36.5%)	69 (38.1%)		
	YES	18 (9.9%)	28 (15.5%)		

CEA = carcinoembryonic antigen, CR = complete response, DSS = disease specific survival, OS = overall survival, PD = progressive disease, PFI = progress free interval, PR = partial response, SD = stable disease.

**Figure 2.**
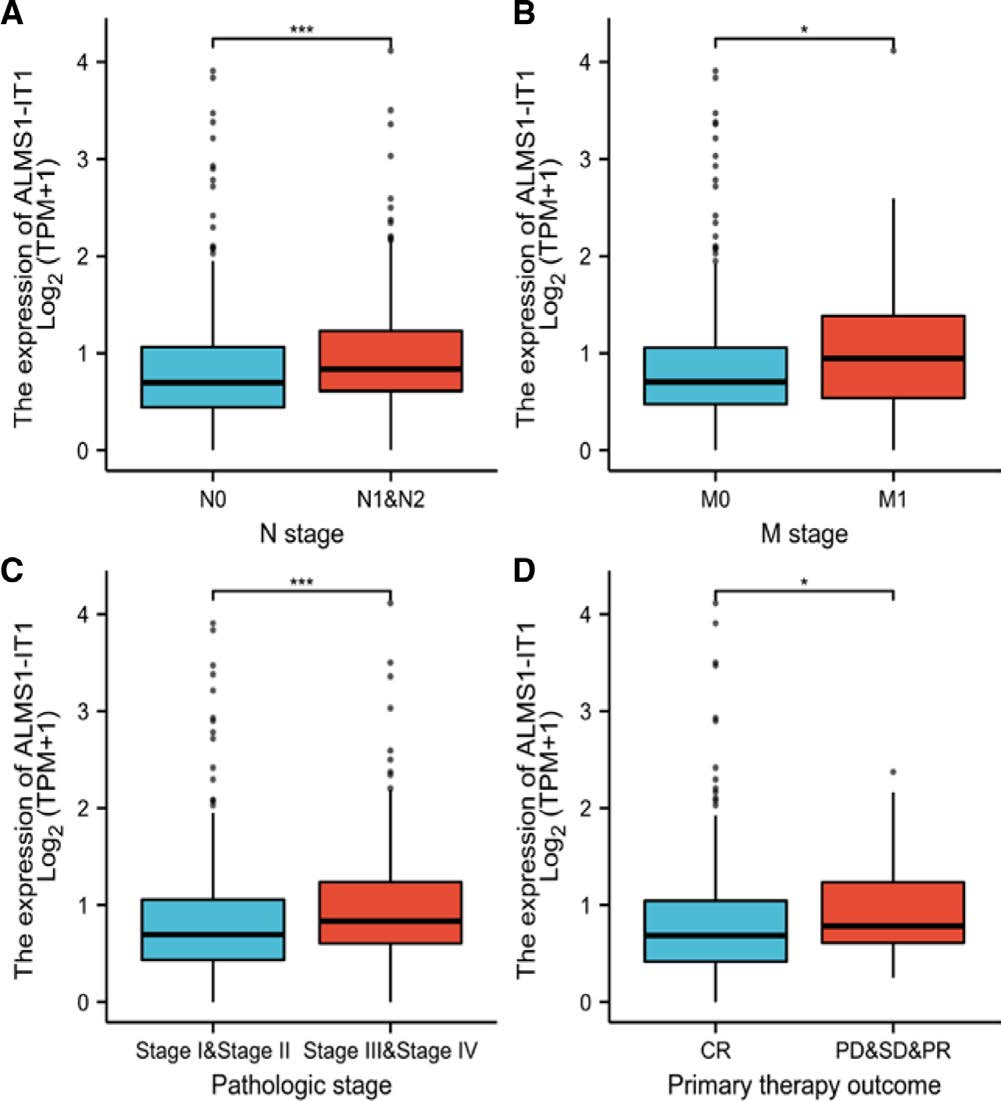
(A) Wilcoxon rank sum test was used to compare the relationship between the expression of ALMS1-IT1 and N stage of COAD patients in TCGA database. (B) Wilcoxon rank sum test was used to compare the relationship between the expression of ALMS1-IT1 and M stage of COAD patients in TCGA database. (C) Wilcoxon rank sum test was used to compare the relationship between the expression of ALMS1-IT1 and Pathologic stage of COAD patients in TCGA database. (D)Wilcoxon rank sum test was used to compare the relationship between the expression of ALMS1-IT1 and primary therapy outcome of COAD patients in TCGA database. ALMS1-IT1 = ALMS1 intronic transcript 1, COAD = colon adenocarcinoma, CR = complete response, PD = progressive disease, SD = stable disease, PR = partial response, TCGA = the cancer genome atlas.

Logistic regression method was also used to show the relationship between the clinicopathological characteristics of COAD and expression level of ALMS1-IT1. The results suggested that ALMS1-IT1 was significantly related to N stage (*P* < .001), M stage (*P* = .002), Pathologic stage (*P* < .001), primary therapy outcome (*P* < .001) (Table 2 and Fig. 2).

**Table 2:** ALMS1-IT1 expression associated with clinicopathologic characteristics (logistic regression).

Characteristics	Total(N)	Odds ratio (OR)	*P* value
T stage (T3&T4 vs T1&T2)	477	1.541 (0.979–2.446)	.063
N stage (N1&N2 vs N0)	478	1.878 (1.299–2.726)	<.001
M stage (M1 vs M0)	415	2.340 (1.364–4.105)	.002
Pathologic stage (Stage III&Stage IV vs Stage I&Stage II)	467	1.968 (1.359–2.862)	<.001
Primary therapy outcome (CR vs PD&SD&PR)	250	0.479 (0.241–0.935)	.033
Lymphatic invasion (YES vs NO)	434	1.196 (0.813–1.763)	.363
Residual tumor (R1&R2 vs R0)	374	1.860 (0.856–4.204)	.122
Colon polyps present (YES vs NO)	249	1.375 (0.809–2.362)	.243
CEA level (>5 vs <=5)	303	1.353 (0.844–2.174)	.210

CEA = carcinoembryonic antigen, CR = complete response, PD = progressive disease, PR = partial response, SD = stable disease.

### 3.2. ALMS1-IT1 expression is correlated with poor prognosis of patients with COAD

**Figure 3.**
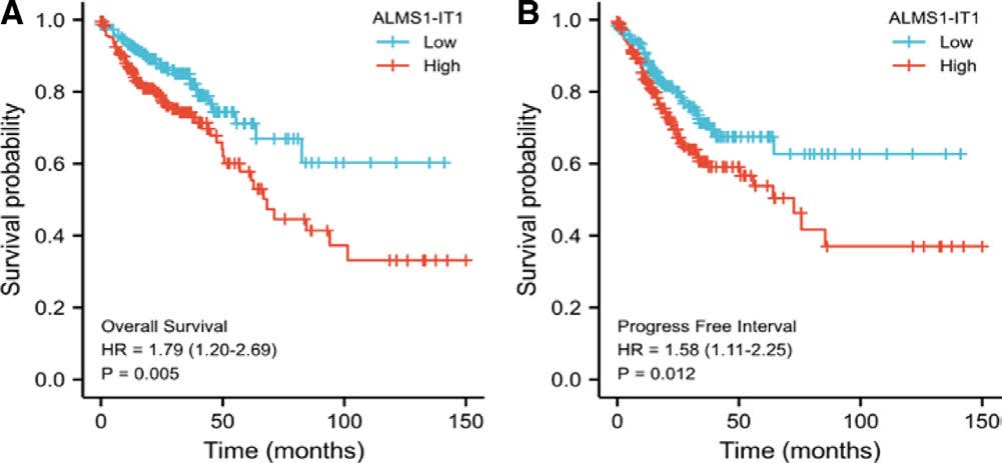
Kaplan–Meier curve was drawn using the R to evaluate the prognostic value of ALMS1-IT1 in OS and PFI of COAD patients. ALMS1-IT1 expression value was divided into high and low expression group according to median value. ALMS1-IT1 = ALMS1 intronic transcript 1, COAD = colon adenocarcinoma, OS = overall survival, PFI = progression-free interval.

The association between ALMS1-IT1 expression and OS or PFI of patients with COAD was evaluated by Kaplan–Meier analysis, which indicated that expression of ALMS1-IT1 is positively correlated with poor OS (*P* = .005, Fig. 3A) and poor PFI (*P* = .012, Fig. 3B) of COAD patients.

### 3.3. ALMS1-IT1 related signaling pathways based on GSEA

**Figure 4.**
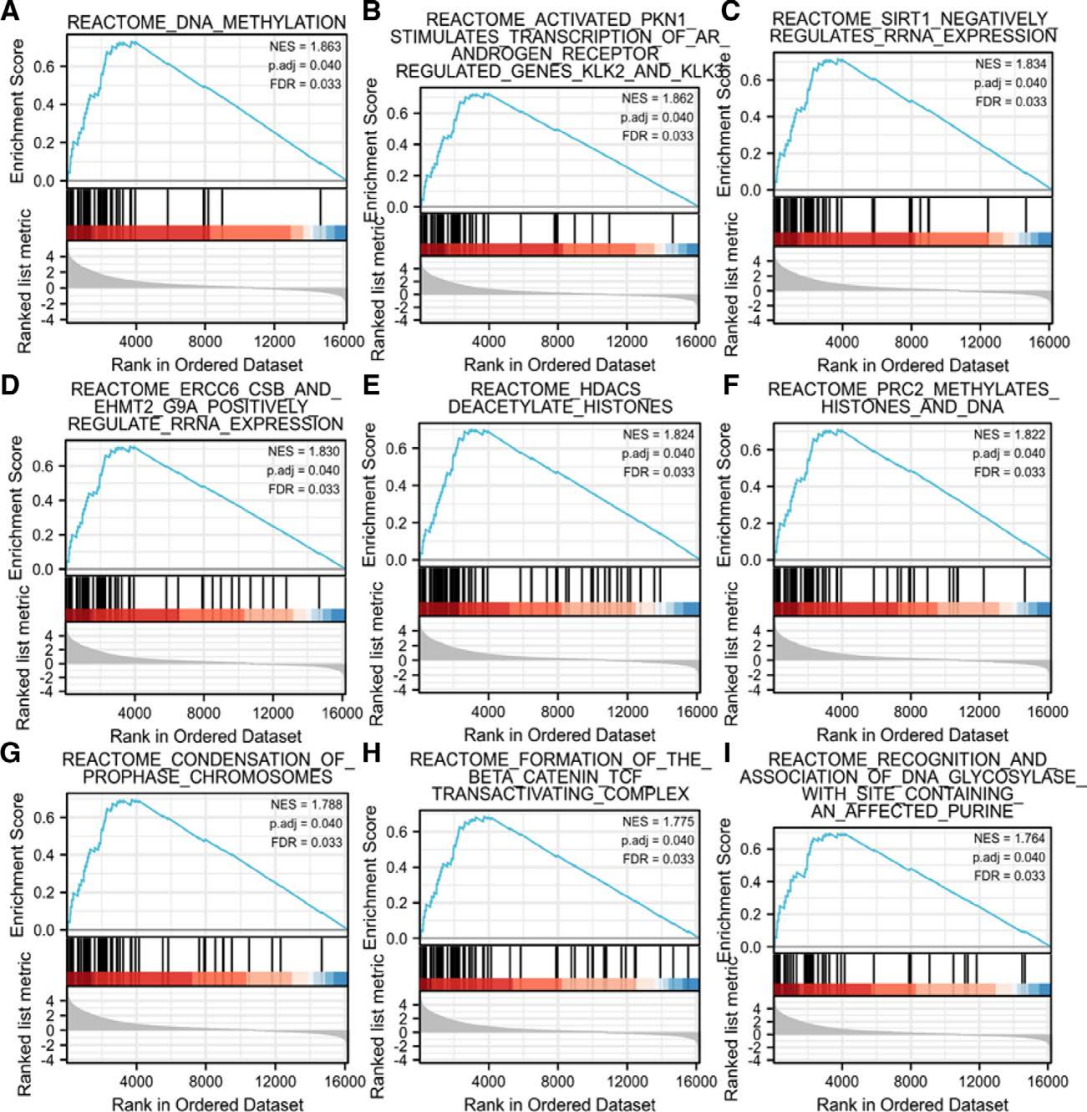
The data set was on the left significantly enriched in red area (ALMS1-IT1 high expression group). NES, normalized NS; Padj, adjust *P* value; ALMS1-IT1 = ALMS1 intronic transcript 1, FDR = false discovery rate.

GSEA was used to identify ALMS1-IT1-related signaling pathways. GSEA revealed significant differences (Padj < 0.05, false discovery rate < 0.25) in enrichment of MSigDB Collection (c2.cp.v7.2.symbols.gmt). We selected the top 9 data sets with high value of normalized enrichment score (Table 3 and Fig. 4).

**Table 3:** REACTOME terms enriched in high- and low-ALMS1-IT1 groups by using GSEA.

ID	NES	p.adjust	FDR
REACTOME_DNA_METHYLATION	1.863	0.040	0.033
REACTOME_ACTIVATED_PKN1_STIMULATES_TRANSCRIPTION_OF_AR_ANDROGEN_RECEPTOR_REGULATED_GENES_KLK2_AND_KLK3	1.862	0.040	0.033
REACTOME_SIRT1_NEGATIVELY_REGULATES_RRNA_EXPRESSION	1.834	0.040	0.033
REACTOME_ERCC6_CSB_AND_EHMT2_G9A_POSITIVELY_REGULATE_RRNA_EXPRESSION	1.830	0.040	0.033
REACTOME_HDACS_DEACETYLATE_HISTONES	1.824	0.040	0.033
REACTOME_PRC2_METHYLATES_HISTONES_AND_DNA	1.822	0.040	0.033
REACTOME_CONDENSATION_OF_PROPHASE_CHROMOSOMES	1.788	0.040	0.033
REACTOME_FORMATION_OF_THE_BETA_CATENIN_TCF_TRANSACTIVATING_COMPLEX	1.775	0.040	0.033
REACTOME_RECOGNITION_AND_ASSOCIATION_OF_DNA_GLYCOSYLASE_WITH_SITE_CONTAINING_AN_AFFECTED_PURINE	1.764	0.040	0.033

NES = normalized enrichment score, FDR = false discovery rate.

### 3.4. ALMS1-IT1 related GO and KEGG analysis

**Figure 5.**
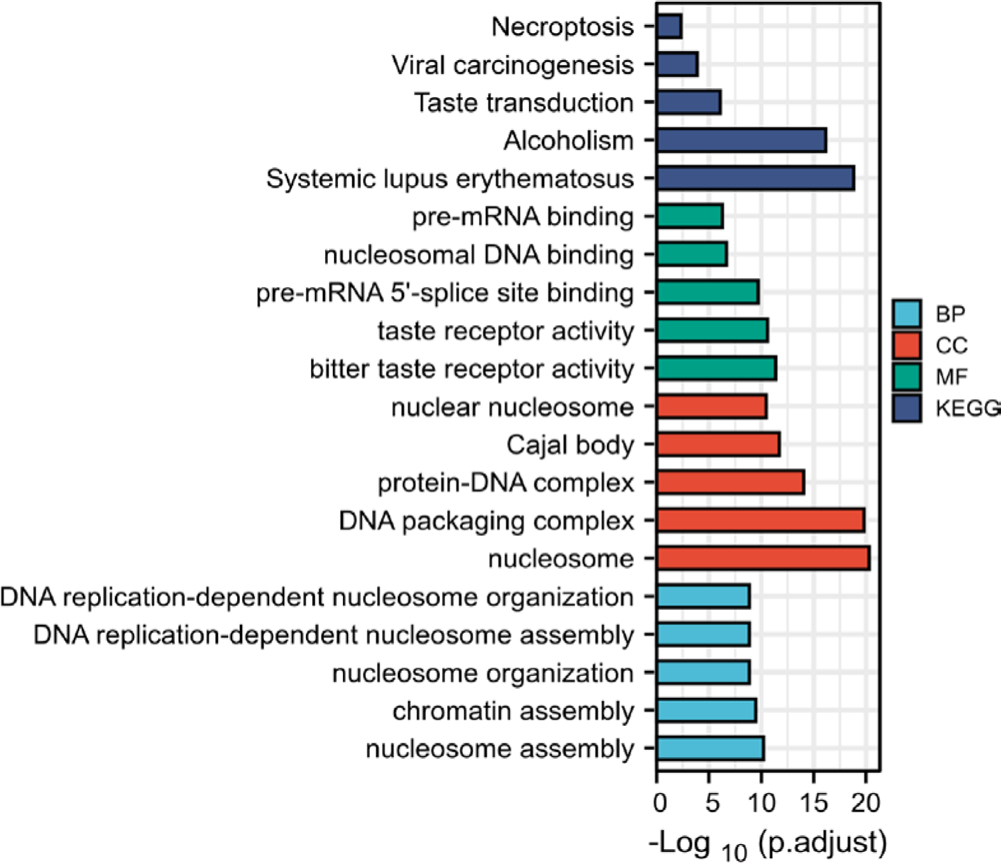
Top 5 of the lowest adj. *P* value of GO and KEGG pathway enrichment analysis of 3303 DEGs related to ALMS1-IT1 in TCGA-COAD data. BP = biological process, CC = cellular component, COAD = colon adenocarcinoma, DEGs = different expression genes, GO = gene oncology, KEGG = Kyoto encyclopedia of genes and genomes, MF = molecular function, TCGA = the cancer genome atlas.

To estimate the potential functions of DEGs in high-risk versus (vs) low-risk groups, we identify DEGs of ALMS1-IT1 in TCGA-COAD data under cutoff criteria of adjusted *P* value <.05 and |logFC|>2.KEGG pathway and GO annotation were performed by R package clusterProfiler(3.14.3).GO reveals the catalogs of biological process, cellular component, and molecular function.After multiple-test correction, KEGG pathways and GO terms with corrected *P* (P.adjust) value <.05 were considered to be prominently enriched in DEGs. We selected top 5 of the lowest adj. *P* value of GO and KEGG pathway enrichment analysis of 3303 DEGs related to ALMS1-IT1 in TCGA-COAD data (Table 4 and Fig. 5).

**Table 4:** GO functional annotation and KEGG pathway analysis.

ONTOLOGY	ID	Description	p.adjust
BP	GO:0006334	nucleosome assembly	6.21275E-11
BP	GO:0031497	chromatin assembly	3.35087E-10
BP	GO:0034728	nucleosome organization	1.40618E-09
BP	GO:0006335	DNA replication-dependent nucleosome assembly	1.40618E-09
BP	GO:0034723	DNA replication-dependent nucleosome organization	1.40618E-09
CC	GO:0000786	nucleosome	5.02992E-21
CC	GO:0044815	DNA packaging complex	1.59647E-20
CC	GO:0032993	protein-DNA complex	9.27504E-15
CC	GO:0015030	Cajal body	1.96108E-12
CC	GO:0000788	nuclear nucleosome	3.34381E-11
MF	GO:0033038	bitter taste receptor activity	4.4215E-12
MF	GO:0008527	taste receptor activity	2.61483E-11
MF	GO:0030627	pre-mRNA 5’-splice site binding	1.98258E-10
MF	GO:0031492	nucleosomal DNA binding	2.05065E-07
MF	GO:0036002	pre-mRNA binding	4.97811E-07
KEGG	hsa05322	Systemic lupus erythematosus	1.57037E-19
KEGG	hsa05034	Alcoholism	7.34901E-17
KEGG	hsa04742	Taste transduction	8.33565E-07
KEGG	hsa05203	Viral carcinogenesis	0.000127823
KEGG	hsa04217	Necroptosis	0.004643185

BP = biological process, CC = cellular component, MF = molecular function.

### 3.5. The correlation between ALMS1-IT1 expression and immune infiltration

**Figure 6.**
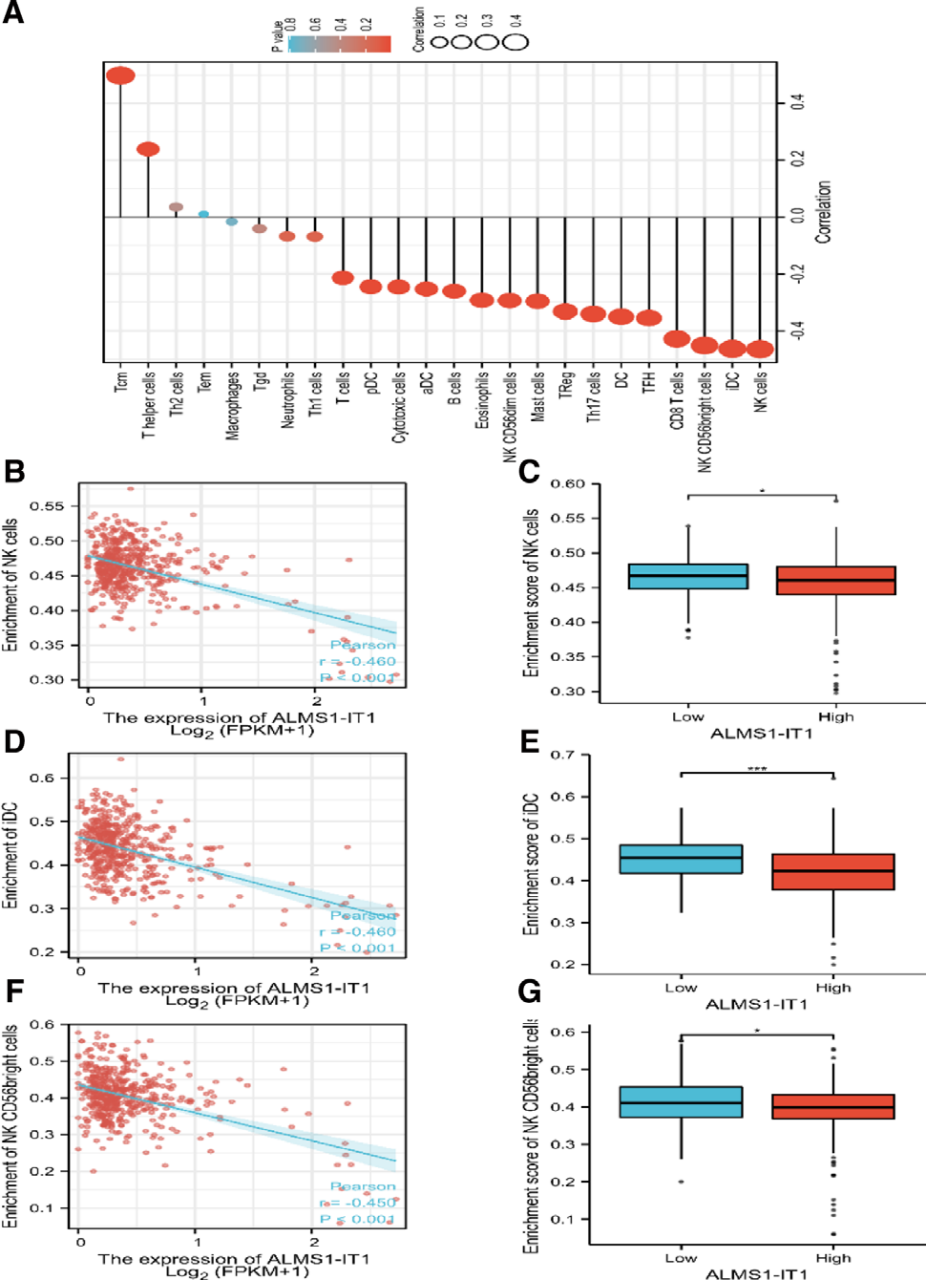
(A) The forest plot shows the correlation between ALMS1-IT1 expression level and 24 immune cells. (B) The correlation between ALMS1-IT1 expression and NK was detected by Pearson correlation method. (C) The Wilcoxon rank sum test was used to analyze the difference of NK infiltration level between ALMS1-IT1 high and low expression groups. (D) The correlation between ALMS1-IT1 expression and iDC was detected by Pearson correlation method. (E) The Wilcoxon rank sum test was used to analyze the difference of iDC infiltration level between ALMS1-IT1 high and low expression groups. (F) The correlation between ALMS1-IT1 expression and NK CD56bright cells was detected by Pearson correlation method. (G) The Wilcoxon rank sum test was used to analyze the difference of NK CD56bright cells infiltration level between ALMS1-IT1 high and low expression groups. ALMS1-IT1 = ALMS1 intronic transcript 1, iDC = immature dendritic cells, Tcm = T central memory, Tem = T effector memory, Tgd = T gamma delta, Tfh = T follicular helper, NK = natural killer, pDCs = plasmacytoid dendritic cells.

We further analyzed the correlation between expression of ALMS1-IT1 and immune infiltration by ssGSEA with Pearson. The results showed that the expression of ALMS1-IT1 was negatively correlated with most immune cells, and the top 3 negative correlation coefficients were natural killer (NK) cells, immature dendritic cell (iDC) and NK CD56bright cells (*P* < .001, Fig. 6).

### 3.6. Target miRNA and protein prediction

**Figure 7.**
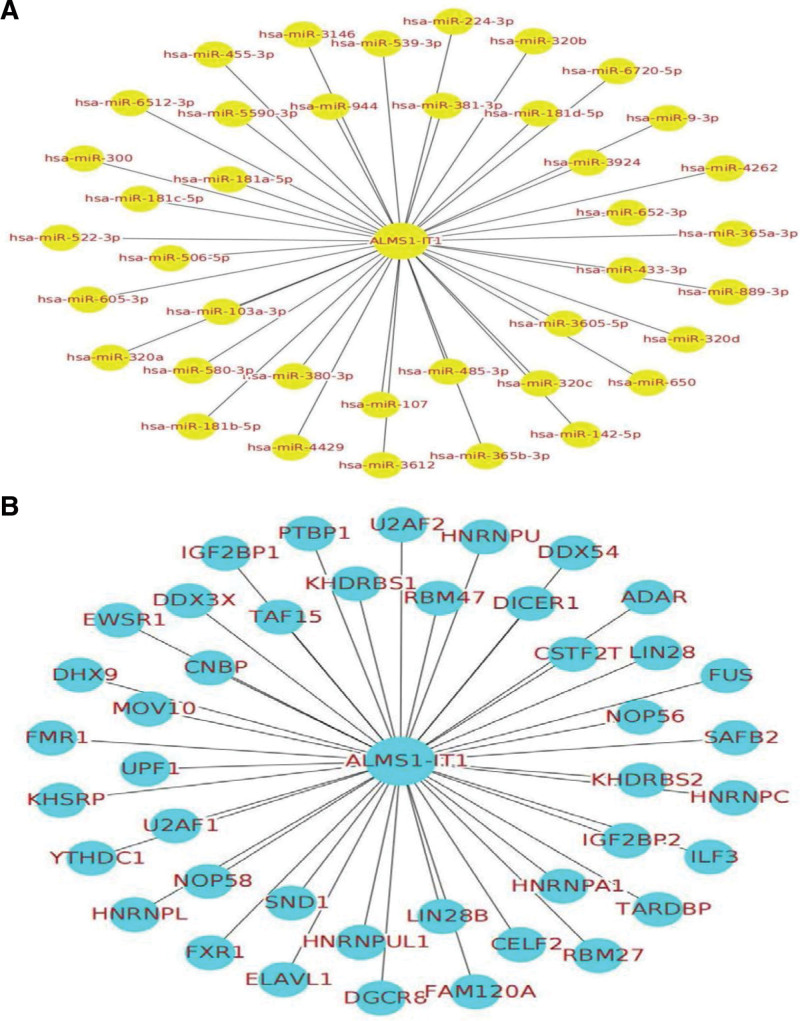
To investigate the lncRNA-miRNA interaction network regulated by ALMS1-IT1, the 39 target miRNAs were identified using Starbase database (A). As for the lncRNA-protein network, the Starbase database was searched and the results revealed that there were 42 RBPs that interacted with ALMS1-IT1 (B). ALMS1-IT1 = ALMS1 intronic transcript 1, lncRNA = long non-coding RNA, RBPs = RNA binding proteins.

Thirty-nine miRNAs and 42 RBPs that may interact with ALMS1-IT1 were identified using Starbase database (Fig. 7).

## 4. Discussion

In this study, the expression of LncRNA ALMS1-IT1 in COAD and its correlation with COAD diagnosis and prognosis were explored. In general, lncRNAs exert regulatory functions at different levels of gene expression, including chromatin modification, transcription, and post-transcription.^[29]^ lncRNAs can interact with chromatin remodeling complexes to induce heterochromatin formation at specific genomic sites and reduce gene expression. In addition, lncRNAs interact with RNA-binding proteins and transcription factor co-activators, or regulate transcription by regulating the main promoters of their target genes. Mechanically, LncRNAs can communicate with DNA, mRNAs, ncRNAs and proteins and play cancer-related regulatory roles, such as signals, decoys, scaffolds and guidelines.^[30,31]^ In addition, lncRNAs were often involved in different stages of CRC, from precancerous polyps to distant metastasis, which can be regarded as potential effective diagnostic biomarkers.^[32,33]^

ALMS1-IT1 is a recently discovered lncRNA, which has been shown to play a key role in regulating tumor progression and predicting the survival time of tumor patients.^[34]^ Luan’s study points out that ALMS1-IT1 was highly expressed in LUAD, and the high expression of ALMS1-IT1 lead to poor prognosis in LUAD patients. Importantly, overexpression of ALMS1-IT1 helps to promote the viability of LUAD cells in vitro.^[22]^ Lei Y’s^[35]^ reveals the significance of the interaction between lncRNAs and ceRNAs in small cell lung cancer, indicating that the integration of expression profiles and alternative splicing can be used to identify biomarkers and potential pathological changes, and ALMS1-IT1 is one of the critical gene. Lu Xing et al^[21]^ reported that ALMS1-IT1 is up-regulated in high-risk groups of head and neck squamous cell carcinoma (HNSCC), which is related to the poor prognosis of HNSCC patients. In addition, it was also found that ALMS1-IT1 is a lncRNA targeting most miRNAs and proteins in HNSCC. All these studies suggest that ALMS1-IT1 may play different roles in various cancer types.

The present study demonstrated the elevated level of ALMS1-IT1 in COAD tissues, which is associated with poor patient outcome. A highlight of this work is to predict the potential mechanisms by which ALMS1-IT1 regulates the development of COAD. Through GO and KEGG, ALMS1-IT1 related gene were found to be involved in nucleosome assembly, chromatin assembly and DNA complex formation, indicating that ALMS1-IT1 may play a role in cell replication. Through GSEA,ALMS1-IT1 was found related in DNA methylation and histone methylation, indicating that ALMS1-IT1 may play a role in the maintenance of cell metabolism and nucleic acid modification and protein modification.

Another important aspect of this study is to investigate the relationship between ALMS1-IT1 expression and diverse immune infiltration levels in COAD. Our results revealed a moderate relationship between ALMS1-IT1 expression and infiltration level of NK cells, iDC and NK CD56 bright cells in COAD.

These correlations could be indicative of a potential mechanism by which ALMS1-IT1 inhibits the function of NK cells, NK CD56 bright cells and iDC, subsequently promotes the function of T central memory, and thus exerts its inhibitory effect on COAD. To our knowledge, despite some limitations, this is the first work to explore the relationship between ALMS1-IT1 and COAD. First of all, the current research is mainly based on bioinformatics analysis, which can be further strengthened through experimental research. Second, the number of healthy subjects as controls is very different from the number of cancer patients. Finally, retrospective research still has its limitations, especially the inconsistent intervention measures and lack of relevant information. Therefore, follow-up studies are needed to further verify our findings.

## 5. Conclusions

Collectively, we observed increased ALMS1-IT1 in COAD, which was also related to poor OS and poor PFI. Moreover, ALMS1-IT1 might participate in the development of COAD via affecting the function of DNA and immune infiltrating cells. The current study partially unveiled the roles of ALMS1-IT1 in COAD and provided a potential biomarker for the diagnosis and prognosis of COAD.

This study was supported by Xiamen medical and health guidance project (No.3502Z20209072).

Xiantao Academic provides technical support for R analysis.

## Author contributions

**Formal analysis:** Ying Li, Zhongying Zhang.

**Methodology:** Yuning Lin, Ying Li, Yongquan Chen, Zhongying Zhang.

**Writing – original draft:** Yuning Lin.

**Writing – review & editing:** Yuning Lin, Ying Li, Yongquan Chen, Zhongying Zhang.
